# Measuring enjoyment of physical activity in older adults: invariance of the physical activity enjoyment scale (paces) across groups and time

**DOI:** 10.1186/1479-5868-8-103

**Published:** 2011-09-27

**Authors:** Sean P Mullen, Erin A Olson, Siobhan M Phillips, Amanda N Szabo, Thomas R Wójcicki, Emily L Mailey, Neha P Gothe, Jason T Fanning, Arthur F Kramer, Edward McAuley

**Affiliations:** 1 Department of Kinesiology and Community Health, University of Illinois at Urbana-Champaign, 906 S. Goodwin Avenue, Urbana, IL 61801, USA; 2 Beckman Institute, University of Illinois at Urbana-Champaign, 405 North Mathews Avenue, Urbana, Illinois 61801, USA

## Abstract

The purpose of this study was to validate the Physical Activity Enjoyment Scale (PACES) in a sample of older adults. Participants within two different exercise groups were assessed at two time points, 6 months apart. Group and longitudinal invariance was established for a novel, 8-item version of the PACES. The shortened, psychometrically sound measure provides researchers and practitioners an expedited and reliable instrument for assessing the enjoyment of physical activity.

## Background

Enjoyment is both a predictor and outcome of physical activity participation [1-3]. Expected enjoyment from physical activities can increase exercise intentions [4] and the mere anticipation of positive emotions predicts physical activity adoption and maintenance [5]. Moreover, stronger anticipation of negative emotions is associated with weaker physical activity intentions and behavior [6]. Although enjoyment has been assessed in numerous studies, no measures of enjoyment have been appropriately validated for use with adult populations. Instead, "measurement equivalence" [7] is often assumed, a pervasive problem associated with many self-report instruments. Measurement equivalence refers to the assumption that a measure has the same meaning across different groups of people (i.e., group invariance), and that its items have the same meaning to individuals across time (i.e., longitudinal invariance). However, it is entirely plausible that questionnaire items hold different meaning to different groups, or that the meaning of items could change across measurement time-points. Each situation would threaten group and longitudinal invariance, two psychometrics properties that are essential in order for researchers and clinicians to draw meaningful interpretations of enjoyment scores.

Little is known about the development of physical activity enjoyment among older adults. Within the interactionist framework of social cognitive theory (SCT) [8,9], self-efficacy beliefs and social factors interact to influence the self-monitoring of one's behavior, its determinants, and its effects. From the perspective of SCT, perceived enjoyment and social support should contribute to the self-regulation of exercise behavior [10]. Additionally, researchers [11,12] have suggested that experienced changes and satisfaction with those changes should result in more positive affective responses over time, which in turn should positively impact future exercise behavior. To date, however, older adults' affective responses to physical activity experiences have mainly been studied in terms of in-task relationships, such as their responses to graded-exercise testing conducted within a laboratory setting [12,13]. However, the enjoyment older adults feel towards the domain of physical activity in general, and its antecedents and consequences, is relatively unexplored. Often, it is assumed that regular exercise is "intrinsically-motivated" but the benefit experienced from one's exercise efforts coupled with support from others may play a more important role in physical activity participation.

The objective of this study was to examine the validity and psychometric properties of the most commonly used measure of enjoyment, the *Physical Activity Enjoyment Scale *(PACES) [14] among a sample of older adults involved in a yearlong exercise program. A secondary purpose was to evaluate the construct validity of the scale with other theoretically-relevant constructs, including perceived social support, experienced exercise-related changes and behavior. The original 18-item PACES was developed by Kendzierski and DeCarlo [14] for a college-age population, and was intended to be uni-dimensional, but further testing in other populations revealed problems with its factor structure [15]. Motl and colleagues [16] used a 16-item version, revised for adolescent girls, which has also been modified for use with younger children [17]. An abbreviated 8-item version of the PACES has been used with adults of mixed ages [2,18] and was found to be invariant across samples of adult runners and cyclists [19]; however, this sample [19] consisted of mostly young and middle-age adults, who have been shown to differ from older adults in their motives for physical activity [20] and perceived experiences of emotion [13,21]. The full 18-item 1-factor structure of the PACES has only been evaluated in one study [22], and again, this study collapsed multiple age groups together, ranging in age from 25 to 75. Together, these findings call for a validation study of the PACES in a sample of older adults.

To date, no version of the PACES has been tested for longitudinal invariance. Without establishing longitudinal invariance, it is difficult to ascertain whether changes in the PACES, or lack thereof, may be attributable to true effects (e.g., intervention, developmental), or to the effects of an unstable, time-dependent measure. Interestingly, Rhodes and colleagues [23] have shown that many interventions designed to change affect, as measured by the PACES, have been ineffective. It is possible, however, that the psychometric properties of the PACES, and other affect scales, are unstable, which could lead researchers to draw false conclusions about any relationships with physical activity. Therefore, one should be cautious in making any interpretations regarding findings based on scales without establishing first that the scale is consistent across groups and time.

Some researchers have claimed that the original 18-item PACES contains questions pertaining to "antecedents and consequences" of the exercise experience [15], two aspects that might vary with time or could even conflict with each other. However, with an invariant measure of enjoyment, we would expect certain relationships between enjoyment and specific theoretically-based antecedents and consequences. Enjoyment has been positively associated with social support, as friends, family, and professionals can enhance physical activity experiences by providing instrumental, informational, emotional, and motivational support [24]. Perceived social support has also been shown to predict exercise behavior indirectly through affect and self-efficacy [25,26]. A meta-analysis [27] found a substantial effect of important others on exercise affect (ES = .63). Thus, as a means of evaluating convergent validity, we examined bivariate associations between our final PACES measure and social support, perceived change brought about by physical activity, and self-reported physical activity.

The purpose of this study was to systematically examine the psychometric properties of the PACES. Group invariance, longitudinal invariance, and convergent validity (with types of perceived social support, experienced exercise-related changes and behavior) were evaluated in a sample of older adults involved in a randomized controlled trial. Thus, we tested the feasibility of two, 1-factor models of PACES (i.e., 18-item and 8-item versions) currently being used in the literature. An alternative, theoretically-based shorter version of the scale was also constructed.

## Methods

### Participants

One-hundred and seventy-nine sedentary older adults (see Table 1 for descriptives) were recruited to participate in a 12-month, two-arm randomized controlled trial. The larger study's primary outcomes were cognitive, brain structure, brain function, and physical functional change and limitations (see [28,29]). The present study involved an analysis of secondary, psychosocial outcomes collected at 6 (*n *= 151) and 12 months (*n *= 146); note that the PACES asks "how you feel at the moment about the physical activity you've been doing", thus it would not have been appropriate to assess PACES at baseline. The study was approved by the university institutional review board at the University of Illinois at Urbana-Champaign, and all inclusion/exclusion criteria relative to study eligibility have been previously described [30].

**Table 1:** Sample Characteristics

Variable	Mean (SD)/%
Age	66.43 (5.67)

Gender	117 (65.4%) Females
	62 (34.6%) Males

Education	20.7% high school graduate
	27.9% some college
	19.6% college graduate
	31.9% graduate degree

Marital Status	59.8% married
	17.9% divorced/separated
	14.0% widowed
	6.7% single
	1.7% partnered/significant other

Race/Ethnicity	88.3% White/Non-Hispanic
	8.4% Black/African-American
	3.4% Asian

### Procedure

Participants who had passed the screening protocol, signed the informed consent, and received medical clearance were scheduled for baseline testing and mailed a psychosocial questionnaire packet. After baseline testing and assessments were complete, all participants were randomly assigned into one of two exercise intervention groups: walking or flexibility-toning-balance (FTB). Both groups exercised three days a week for approximately one hour, and differed mainly in their mode of exercise. The walking group engaged in distance-walking at specified intensities, whereas the FTB group engaged in a variety of age-appropriate flexibility, strength, and balance training exercises (see [30,31] for further details).

## Measures

### Demographics

Age, gender, education, and marital status were assessed.

### Physical Activity Enjoyment Scale

The original 18-item PACES [14] scale was used to assess enjoyment. Respondents were asked to rate "how you feel at the moment about the physical activity you have been doing" using a 7-point bipolar rating scale. Eleven items are reverse scored. Higher PACES scores reflect greater levels of enjoyment.

### Social Support

Social support was assessed with the Social Provisions Scale (SPS) [32]. The SPS is a 24-item scale with six subscales (i.e., attachment, social integration, reassurance of worth, reliable alliance, opportunity for nurturance, and guidance), each consisting of four items. The scale has been shown to be invariant across time in a sample of older adults [33]. Higher SPS scores reflect greater levels of social support.

### Perceived Change

We used a 14-item Likert scale measure (1 = Much Worse, 3 = No Difference, 5 = Much Better) of perceived *physical *(7 items; e.g., joint pain, flexibility), *emotional/psychological *(4 items; e.g., attitude toward physical activity, support for physical activity from group or family members), and *functional *(3 items; e.g., difficulty getting out of a car or rising from a seated position, going up and down stairs) change brought about by physical activity.

### Self-reported Physical Activity

The *Physical Activity Scale for the Elderly *(PASE; [34]), was used to assess physical activity behavior. Participants reported the frequency they participated in leisure activities (e.g., outdoor walking, light, moderate, and strenuous sport and recreation, and muscle strengthening) by indicating never, 1-2 days/week (seldom), 3-4 days/week (sometimes), or 5-7 days/week (often). Activity duration was indicated as either less than 1 hour, between 1-2 hours, 2-4 hours, or more than 4 hours. Items were summers after being weighted with values determined by prior validation studies with older adults [35].

### Data Analysis

Models were sequentially tested using *M*plus version 6.0 [36]. We initially assessed the structural validity of a 1-factor PACES model using confirmatory factor analysis (CFA) with a robust maximum likelihood estimator (MLR). Multiple data fit indices were considered in the detection of model misspecification, including the chi-square statistic (χ^2^), a test of exact model-to-data fit was used (significant *p *values indicate improper model specification), the root mean square error of approximation (RMSEA; cutoff value of < .06 has been recommended [37] and indicates good fit) the comparative fit index (CFI) and Tucker-Lewis Index (TLI). CFI and TLI values ≥ .95 have been suggested [37,38] and indicate excellent fit. Fit indices are sensitive to sample size and model type [39-41], thus we have reported multiple criteria, as recommended by the majority of psychometricians [37,39,40].

### Invariance Testing

**Figure 1:**
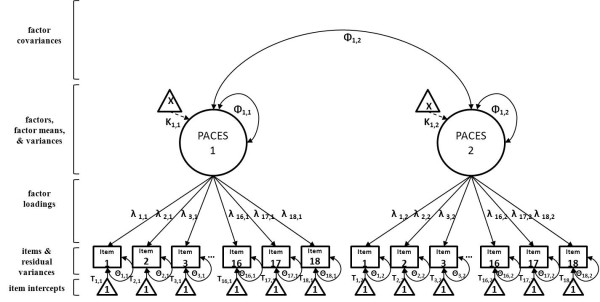
**Model Parameters Involved in Testing Invariance of 8-item PACES**.

After structural validity at time 1 was determined, group invariance and longitudinal invariance were examined. Invariance testing involves the sequential comparison across nested models through the incremental addition of equality constraints on model parameters (see Figure 1 for a graphical depiction of model parameters). For group invariance tests, equality constraints were successively added for model parameters between Walking and FTB groups, and for longitudinal invariance, equality constraints were added for model parameters between time 1 and 2 (groups were collapsed). The procedure is equivalent for testing group and longitudinal invariance. First, one must test equivalence of the factor structure itself (i.e., configural invariance), followed by the equivalence of the factor loadings (i.e., metric invariance), intercepts (scalar invariance), and then residual variances (strict invariance). We set the metric of the scale by constraining the factor means to zero and factor variances to 1. Additionally, we tested invariance of latent factor means and variances across groups and time. Evidence of invariance of parameters between nested models was based on non-significant chi-square difference tests, corrected for non-normality [42], along with change in CFI < .01 [43] and RMSEA < .015 [44]. Model modifications were primarily based on substantive and methodological considerations.

## Results

### Preliminary Analyses

Data were initially analyzed to assess normality assumptions. Responses to items were somewhat negatively skewed, therefore the MLR estimator in *Mplus *was used in all subsequent modeling. Full-information estimation was used for missing data [45]. There was 0% missing data at Time 1 and 2.67% missing data for the raw scores at Time 2.

### Psychometric Evaluation of the PACES

#### Structural Validity

Kendzierski and DeCarlo's [14] original 18-item, one-dimensional model (see Table 2 for entire list of items) provided a poor fit to the data at baseline. Specifically, χ^2^, RMSEA, CFI, and TLI suggested severe misspecification of the model (χ^2 ^= 327.833(135), *p *< .001, RMSEA = .097 [95% CI = .084, .111], CFI = .848, TLI = .828). Due to the negatively-worded items (11 of 18 items), it is quite possible that method effects may account for significant variance in the model [46,47]. Following Marsh et al's [46] procedure, we systematically evaluated a series of parameter modifications including: correlated uniquenesses (CU) among positively-worded items (χ^2 ^= 227.389(114), *p *< .001, RMSEA = .081 [95% CI = .066, .096], CFI = .911, TLI = .880), a positive latent method factor (LMF) (χ^2 ^= 243.887(128), *p *< .001, RMSEA = .077 [95% CI = .063, .092], CFI = .909, TLI = .891), CU among negatively-worded items (χ^2 ^= 124.368(80), *p *= .001, RMSEA = .061 [95% CI = .039, .081], CFI = .965, TLI = .933), a negative LMF (χ^2 ^= 254.763(124), *p *< .001, RMSEA = .084 [95% CI = .069, .098], CFI = .897, TLI = .873), and both a positive LMF and a negative LMF (χ^2 ^= 195.493(117), *p *< .001, RMSEA = .067 [95% CI = .050, .083], CFI = .938, TLI = .919). No further modifications were deemed appropriate and the full18-item model was dropped from further analyses.

**Table 2:** Physical Activity Enjoyment Scale (PACES) 18-items

#	Item
1	I enjoy it; I hate it
2	I feel bored; I feel interested
3	I dislike it; I like it
4	I find it pleasurable; I find it unpleasurable
5	I am very absorbed in this activity; I am not at all absorbed in this activity
6	It's no fun at all; It's a lot of fun
7	I find it energizing; I find it tiring
8	It makes me depressed; It makes me happy
9	It's very pleasant; It's very unpleasant
10	I feel good physically while doing it; I feel bad physically while doing it
11	It's very invigorating; It's not at all invigorating
12	I am very frustrated by it; I am not at all frustrated by it
13	It's very gratifying; It's not at all gratifying
14	It's very exhilarating; It's not at all exhilarating
15	It's not at all stimulating; It's very stimulating
16	It gives me a strong sense of accomplishment; It does not give me any sense of accomplishment
17	It's very refreshing; It's not at all refreshing
18	I felt as though I would rather be doing something else; I felt as though there was nothing else I would rather be doing

Raedeke's [18] 8-item measure (i.e., items 1, 2, 3, 4, 5, 6, 9, and 18) was examined next. This model provided a slightly better fit than the unmodified 18-item model, but still suggested severe misspecification (χ^2 ^= 78.686(20), *p *< .001, RMSEA = .139 [95% CI = .108, .172], CFI = .875, TLI = .825). Several parameterizations accounting for method effects improved fit indices. Specifically, adding CU among only positively-worded items fit the data well (χ^2 ^= 22.301(14), *p *= .073, RMSEA = .063 [95% CI = .000, .109], CFI = .982, TLI = .965) as did the positive LMF approach (χ^2 ^= 26.678(16), *p *= .045, RMSEA = .066 [95% CI = .010, .110], CFI = .977, TLI = .960). The addition of CU among only negatively-worded items (χ^2 ^= 33.860(14), *p *= .002, RMSEA = .097 [95% CI = .056, .139], CFI = .958, TLI = .916) and the negative LMF approach (χ^2 ^= 42.949(16), *p *< .001, RMSEA = .106[95% CI = .068, .144], CFI = .943, TLI = .900) provided a poor fit; however, the model accounting for both a positive LMF and a negative LMF fit the data very well (χ^2 ^= 14.461(11), *p *= .209, RMSEA = .046 [95% CI = .000, .103], CFI = .993, TLI = .981). In sum, this 8-item measure appears to have an inherent response bias due to the fact that half of the items are worded in reverse. Given that out-of-range parameter estimates and other inadmissible solutions were obtained with the best-fitting models (a common problem with LMF models [48]), we opted to search for a more parsimonious, less parameterized model.

### Alternative Model Testing

Due to the problems with both established versions of the PACES, we attempted to validate a novel version by having an expert panel of exercise psychologists re-examine the content of all of the original items. It is well-established that emotional self-report depends on the accessibility of emotions [49,50], and our general framework for selecting a new combination of items was based on evidence that older adults are better than younger adults at "affective balance" [51] and that they strategically regulate emotion by focusing on positive events [52]. This adaptive process could cause older adults to apply their own personal, idiosyncratic theories when judging their emotions. Thus, negatively worded items and those with explicit meaning could be de-contextualized, and in turn, feeling bad, pain, and other physiological and emotional states, may greatly influence their choices on any given day. The expert panel was instructed to select only items referring to eudaimonic (i.e., psychological and social well-being) aspects rather than hedonic (i.e., affective balance and life-fulfilling) aspects of physical activity, aspects of the scale that may be less impacted by fluctuations in feeling states or strategic regulation. This resulted in an alternative, 8-item model (i.e., items 4, 6, 9, 11, 13, 14, 15, 17) 

Several researchers [15,18] have claimed that the original scale contains items that assess the perception of enjoyment and antecedents/consequences of enjoyment. The panel agreed with this logic and removed the item, "It gives me a strong sense of accomplishment". Also, Heesch et al. [22] determined that one item (i.e., "I am very absorbed") provided a poor fit with the overall model and the panel agreed that the item should be removed as the perceived meaning of "absorbed" may be dubious. The panel also felt there was a fundamental problem with the "I enjoy it; I hate it" and "It makes me depressed; It makes me happy" items, as hate and depression are not necessarily on the same spectrum as enjoyment. More appropriate bipolar ratings for these items would parallel the phrasing used in others such as "I *do not *enjoy it" and "It *does not *make me happy." Also, "boredom", "like", "pleasant", and "nothing I'd rather be doing" were removed because the items do not isolate enjoyment and it could be argued that they do not reflect enjoyment at all. Finally, two items (i.e., feeling "frustrated" and "feeling good physically while doing it"), which likely hold different meaning with age and inexperience, were removed due to low relevance for our inactive, older adult sample.

A confirmatory factor analysis showed that this model provided an excellent fit (χ^2 ^= 24.164(20), *p *= .235, RMSEA = .037 [95% CI = .000, .083], CFI = .988, TLI = .983). Note that the modified scale still contains two negatively-worded items, but alternative models did not significantly improve the model fit. Reliability was calculated with standardized estimates using McDonald's omega [53] coefficient (ω = (Σλi)²/([Σλi]²+Σδii) where λi are the factor loadings and δii the error variances. Results revealed good internal reliability coefficients for the new 8-item PACES measure at time 1 and 2 (ω = .93, .93).

## Group Invariance of the PACES-8

### Invariance across exercise groups

To determine if our novel, best-fitting, 8-item model could be generalized across older adults engaging in different modes of exercise, we next tested group invariance across both exercise conditions. The configural model, with each item regressed on a single latent enjoyment factor, fit the data extremely well (χ^2 ^= 32.619(40), *p *= .790, RMSEA = .000 [95% CI = .000, .054], CFI = 1.000, TLI = 1.027). The metric invariance model, with the addition of identical factor loadings across groups, also provided an excellent fit to the data (χ^2 ^= 40.197(47), *p *= .748, RMSEA = .000 [95% CI = .000, .056], CFI = 1.000, TLI = 1.021), and the adjusted Satorra-Bentler (S-B) χ^2 ^Δ test [42] was not significant. Next, the item intercepts were constrained across groups and this scalar invariance model provided a very good fit as well (χ^2 ^= 44.450(54), *p *= .820, RMSEA = .000 [95% CI = .000, .046], CFI = 1.000, TLI = 1.026), and the S-B χ^2 ^Δ test was not significant. Then, residual variances were constrained across groups and this residual invariance model provided a good model-data fit (χ^2 ^= 63.170(62), *p *= .435, RMSEA = .016 [95% CI = .000, .072], CFI = .997, TLI = .997), and the S-B χ^2 ^Δ test was not significant (note that the RMSEA change did exceed the recommended cutoff by .001). We also wanted to test whether any latent mean differences existed in between the two groups. Therefore, we constrained latent factor means to be equal and this model fit the data well (χ^2 ^= 64.390(63), *p *= .428, RMSEA = .017 [95% CI = .000, .072], CFI = .996, TLI = .997), and the S-B χ^2 ^Δ test was not significant. Finally, we constrained the latent variances and this model did not significantly change in fit (χ^2 ^= 64.776(64), *p *= .449, RMSEA = .013 [95% CI = .000, .070], CFI = .998, TLI = .998). Together, these findings suggest that latent mean scores may be compared across groups and that there were no differences in enjoyment. Factor loadings and residuals for the model are reported in Table 3. Due to our small sample size, and given that the alternative PACES model was invariant across exercise groups, we collapsed the sample and retained the model for further invariance testing.

**Table 3:** PACES-8 Factor Loadings and Residuals across Group and Time (Least Restrictive, Most Restrictive)

Group Invariance	Walking (n = 75)		Flexing-Toning-Balance (n = 76)	
**Items**	***Loadings*** **(λ)**	***Residuals*** **(θ)**	***Loadings*** **(λ)**	***Residuals*** **(θ)**

I find it pleasurable	.70, .71	.51, .50	.72, .71	.48, .50
It's a lot of fun^†^	.86, .82	.27, .32	.80, .82	.37, .32
It's very pleasant	.79, .69	.38, .52	.61, .69	.63, .52
It's very invigorating	.80, .83	.36, .32	.84, .83	.30, .32
It's very gratifying	.83, .74	.32, .45	.66, .74	.57, .45
It's very exhilarating	.85, .78	.29, .40	.70, .78	.51, .40
It's very stimulating^†^	.78, .86	.39, .27	.92, .86	.15, .27
It's very refreshing	.80, .84	.36, .30	.88, .84	.23, .30

**Longitudinal Invariance**	**Time 1**		**Time 2**	

**Items**	*Loadings* (λ)	*Residuals* (θ)	*Loadings* (λ)	*Residuals* (θ)

I find it pleasurable	.70, .70	.52, .52	.69, .70	.53, .52
It's a lot of fun^†^	.83, .78	.31, .39	.74, .78	.45, .39
It's very pleasant	.68, .73	.54, .47	.76, .73	.42, .47
It's very invigorating	.83, .86	.32, .25	.90, .86	.19, .25
It's very gratifying	.74, .78	.46, .39	.82, .78	.32, .39
It's very exhilarating	.78, .84	.39, .30	.89, .84	.20, .30
It's very stimulating^†^	.86, .76	.26, .43	.68, .76	.54, .43
It's very refreshing	.83, .85	.31, .27	.86, .85	.26, .27

**Note**. ^† ^Items are reverse coded and were inverted prior to analyses.

### Longitudinal Invariance of the PACES-8

Next, we conducted invariance testing across time. The longitudinal configural invariance for the 8-item measure provided an adequate fit to the data (χ^2 ^= 123.512 (95), *p *= .026, RMSEA = .045 [95% CI = .016, .065], CFI = .972, TLI = .965). The metric invariance model showed little change in overall fit (χ^2 ^= 134.905(102), *p *= .016, RMSEA = .046 [95% CI = .021, .066], CFI = .968, TLI = .962), and the S-B χ^2 ^Δ test was not significant. Scalar invariance also provided similar fit indices (χ^2 ^= 140.879(109), *p *= .022, RMSEA = .044 [95% CI = .018, .064], CFI = .969, TLI = .966), and S-B χ^2 ^Δ was not significant. In addition, the residual invariance model also provided an excellent fit to the data (χ^2 ^= 148.924(117), *p *= .025, RMSEA = .043 [95% CI = .016, .062], CFI = .969, TLI = .968), one which was not significantly different from the less restrictive, scalar model. Finally, constraining latent means (χ^2 ^= 149.288(118), *p *= .027, RMSEA = .042 [95% CI = .015, .061], CFI = .969, TLI = .969) and variances (χ^2 ^= 150.742(119), *p *= .026, RMSEA = .042 [95% CI = .016, .061], CFI = .969, TLI = .969) did not change the fit indices (see Table 3 for factor loadings and residuals). Together, these findings imply that there are no threats to longitudinal invariance for the revised version of the PACES, and the level of enjoyment also apparently did not change.

### Convergent Validity

Bivariate associations (see Table 4) were examined between the new PACES-8 total scores and SPS scales at times 1 and 2. Relationships were significant, positive, and ranged from .18 to .31 (see Table 4), with the exception of the nurturance scale (*p *> .05). The PACES-8 correlated positively with experienced physical change (r's = .42, .47), psychological/emotional change (.41, .42), and functional change (.39, .29) at times 1 and 2, respectively. The correlation between PASE and PACES-8 was marginal (r = .16, *p *= .05) to small (r = .17, *p *= .04). PACES-8 strongly correlated with the original 18-item version at both time points (.98, .97).

**Table 4:** Correlations among PACES-8 and Social Provisions

Measure	1	2	3	4	5	6	7	8	9	10	11	12	13	14	15	16
1. PACES-8 (6 Months)	--															
2. SPS-Total (6 Months)	27**	--														
3. SPS-Guidance (6 Months)	.24**	.86**	--													
4. SPS-Reassurance (6 Months)	.21*	.72**	.60**	--												
5. SPS-Social Integration (6 Months)	.21**	.82**	.66**	.57**	--											
6. SPS-Attachment (6 Months)	.24**	.88**	.75**	.55**	.72**	--										
7. SPS-Nurturance (6 Months)	.11	.52**	.24**	.20*	.24**	.34**	--									
8. SPS-Reliable Alliance (6 Months)	.24**	.78**	.74**	.53**	.60**	.64**	.16	--								
9. PACES-8 (12 Months)	.65**	.31**	.32**	.29**	.27**	.30**	.02	.29**	--							
10. SPS-Total (12 Months)	.28**	.83**	.71**	.62**	.67**	.70**	.41**	.67**	.31**	--						
11. SPS-Guidance (12 Months)	.19*	.69**	.73**	.54**	.59**	.59**	.13	.60**	.28**	.81**	--					
12. SPS-Reassurance (12 Months)	.18*	.54**	.39**	.62**	.44**	.39**	.25**	.40**	.21*	.67**	.40**	--				
13. SPS-Social Integration (12 Months)	.24**	.66**	.54**	.52**	.68**	.56**	.22**	.54**	.28**	.83**	.62**	.61**	--			
14. SPS-Attachment (12 Months)	.25**	.73**	.67**	.45**	.55**	.73**	.26**	.63**	.30**	.82**	.67**	.46**	.63**	--		
15. SPS-Nurturance (12 Months)	.15	.42**	.17*	.20*	.18*	.28**	.74**	.16	.05	.52**	.17*	.22**	.30**	.22**	--	
16. SPS-Reliable Alliance (12 Months)	.25**	.63**	.67**	.45**	.56**	.51**	.08	.69**	.27**	.78**	.72**	.42**	.59**	.62**	.16*	--

**Note**. ** *p *< .001; * *p *< .05.

## Discussion

Enjoyment is consistently reported by older adults as a motive for exercise participation [1,54]. The purpose of this study was to evaluate the psychometric properties (i.e., group and longitudinal invariance) associated with the *Physical Activity Enjoyment Scale*, the field's most widely used measure of participant-reported enjoyment. Without these characteristics, meaningful conclusions cannot be drawn from PACES scores. Moreover, any differences found between groups and across time would imply inherent measurement variability. Consistent with work done by Motl et al. [16] and Moore et al. [17] in younger populations, we found that the original 18-item PACES did not represent a strong 1-factor model for this sample of older adults. Additionally, an 8-item measure that has previously been used with adult samples [18,19] also failed to provide an adequate fit. Ultimately, our expert panel constructed a novel, 8-item measure of enjoyment. Our 8-item version was invariant across two exercise groups, over a 6-month time-frame, which indicates that the new combination of items have strong psychometric integrity.

With our new, internally consistent, group and longitudinally invariant measure of PACES, we examined whether any substantive differences were evident in older adults engaging in two different exercise modalities. Interestingly, the two exercise conditions did not differ in enjoyment. This is not altogether surprising, as aerobic and resistance training have each been associated with favorable self-reported changes in vitality, and less favorable outcomes including reduced pleasure and increased fatigue [13,55-57]. On the other hand, these two training modes produce different patterns of cognitive and brain changes [28,29,58]. It is also possible that *anticipated *positive and negative emotions may have balanced out our participants' overall enjoyment. Despite this, we can infer that both groups experienced sufficient levels of joy given that means were at the midpoint of the scale and overall attendance rates were high (approximately 80%).

As expected, the revised PACES-8 was positively correlated with five of six SPS subscales at both time points. The "opportunity for nurturance" scale did not correlate with enjoyment. Being responsible for others at home may be a value held by older adults, but caring for others in an exercise group may be less relevant. In fact, research by Carstensen and her colleagues [59,60] suggests that over time, older adults are less interested in making new friends and would rather focus on maintaining their established inner circle of friends. This intentional "emotional regulation" may explain the nonsignificant relationship, however, it should be noted that exercising within a group is preferred by some older adults [61], including unmarried adults [62], and these individuals may have in turn, experienced greater enjoyment. Further evidence for convergent validity was demonstrated by positive associations between perceived physical, psychological and functional changes from exercise and enjoyment and a more modest association between enjoyment and self-reported physical activity.

There are some limitations of this study worth noting, including the study's demographic characteristics which consisted primarily of White (91%) females (65%). Older adults from more varied backgrounds may have a different concept of enjoyment. For example, the meaning of enjoyment may be different for older adults who may have had to use physical activity as a means of transportation to work every day. Unfortunately, invariance testing across subgroups was not possible due to limitations in sample size. The study is also limited by the fact that change in enjoyment could not be tested between baseline (inactivity) and subsequent measurements, when we might expect change. This is also a general limitation of the PACES, as it was intended for people already involved in physical activity, and it limits the scale's utility. Finally, we cannot say for sure that age differences are contributing to the problems with the original scale's factor structure, and direct comparisons across age are needed. Future studies should examine the psychometric properties of the scale in other populations across other time-frames (e.g., 12 months) and exercise modalities.

This study has important practical implications for any conclusions based on the original 18-item PACES. In fact, our findings suggest that studies reporting changes based on the 18-item version may reflect a change in measurement characteristics rather than a change in the construct itself. On a positive note, the revised scale is shorter and may expedite clinical assessment and reduce participant burden. Most importantly, this study has validated a measure of enjoyment in older adult populations that is invariant across exercise modality and time, and corresponds with an established measure of perceived social support.

## Conclusions

Enjoyment is an important construct in physical activity participation and maintenance. Accurate and valid measurement is critical for research comparison and integrity. Our results show that an 8-item revised scale is invariant across groups and time in a sample of community-dwelling older adults. These results are consistent with previous work and provide practical applications for future clinical and research use.

## Competing interests

The authors declare that they have no competing interests.

## Authors' contributions

All authors read and approved the final manuscript. SM: manuscript writing, study design, data analysis; EO: manuscript revisions, data analysis; SW: manuscript revisions, data analysis and collection; AS: manuscript revisions, data analysis and collection; TW: manuscript revisions, data analysis and collection; EM: manuscript revisions, data analysis and collection; NG: manuscript revisions, data analysis; JF: manuscript revisions, data analysis; AK: design of the parent study; manuscript revisions; EM: study design, manuscript revisions, supervision of research group
